# Safely Dissolvable and Healable Active Packaging Films Based on Alginate and Pectin

**DOI:** 10.3390/polym11101594

**Published:** 2019-09-29

**Authors:** Maziyar Makaremi, Hosnieh Yousefi, Giuseppe Cavallaro, Giuseppe Lazzara, Calvin Bok Sun Goh, Sui Mae Lee, Atefeh Solouk, Pooria Pasbakhsh

**Affiliations:** 1 Advanced Engineering Platform, Mechanical Engineering Discipline, School of Engineering, Monash University Malaysia, Selangor 47500, Malaysia; 2 Biomedical Engineering faculty, Amirkabir University of Technology (Tehran Polytechnic), Tehran 15875-4413, Iran; 3 Department of Physics and Chemistry, University of Palermo, Viale delle Scienze, pad. 17, 90128 Palermo, Italygiuseppe.lazzara@unipa.it (G.L.); 4 School of Science, Monash University Malaysia, Selangor 47500, Malaysia

**Keywords:** pectin, alginate, biocomposites, food packaging, degradable films

## Abstract

Extensive usage of long-lasting petroleum based plastics for short-lived application such as packaging has raised concerns regarding their role in environmental pollution. In this research, we have developed active, healable, and safely dissolvable alginate-pectin based biocomposites that have potential applications in food packaging. The morphological study revealed the rough surface of these biocomposite films. Tensile properties indicated that the fabricated samples have mechanical properties in the range of commercially available packaging films while possessing excellent healing efficiency. Biocomposite films exhibited higher hydrophobicity properties compared to neat alginate films. Thermal analysis indicated that crosslinked biocomposite samples possess higher thermal stability in temperatures below 120 °C, while antibacterial analysis against *E. coli* and *S. aureus* revealed the antibacterial properties of the prepared samples against different bacteria. The fabricated biodegradable multi-functional biocomposite films possess various imperative properties, making them ideal for utilization as packaging material.

## 1. Introduction

Packaging has rapidly developed in different industries, especially in the food industry, because many foodstuffs are being supplied in packages in developed countries. During the past decades, polymers were used for food packaging extensively because they have advantages in comparison to traditional material (e.g., glass, tin plate) [1]. Plastics have many different compositions which allow designing appropriate packaging for each product specifically [2]. They are also inexpensive, thermo sealable, and low weight materials that are able to print and fit in different shapes [2,3].

In the last few decades, the population growth has increased the use of petroleum based non-biodegradable polymers enormously and this led to an increase in the amount of non-biodegradable waste materials in our environment [4]. A big portion of this waste plastic will end up in the marine and land ecosystem where it suffers degradation and fragmentation [5]. Microplastic debris (<5 mm), such as microcapsules or microbeads proliferate, migrate and accumulate in natural habitats [6,7]. The debris can be dangerous for marine animals in the ocean and also immigrate from the ocean and deposited on beaches [6].

In the past few decades, many studies have been done about biodegradable materials for various applications such as biomedical, packaging, agronomical, and textile industry. This is due to their excellent advantages over non-degradable materials including biodegradation and biocompatibility. They are also processable and eco-friendly [8]. Poly glycolic acid, poly lactic acid, and polydioxanone are the poly(α-hydroxy acid) and most usual synthetic biodegradable polymers [9]. Furthermore, other types of biopolymer that have attracted researchers include cellulose, chitosan, collagen, pectin, and alginate [10].

Pectin is a hetero-polysaccharide composed of (1,4)-α-linked galacturonic acid and (1,2)-linked rhamnose. There are also galactosyl and L-arabinosyl side chains in the pectin structure [11]. It is found in plants which cause strength and pliability and is common in many fruits and vegetables. This polysaccharide has several functions. For example, cell signaling, adhesion, proliferation, and differentiation [12]. Pectin is applicable in foodstuffs production as a stabilizer and gelling ingredient because of its gel-forming ability. Moreover, its good gelling properties, biocompatibility, and biodegradability make it an interesting novel biomaterial for biomedical applications (e.g., pharmaceutic and cosmetic) [13,14]. Pectins can be low-methoxyl or high-methoxyl based on their degree of methylation [15]. Properties of pectin such as gelation, solvability, and film formation are dependent on the degree of methylation [16]. The origin and processing manners determine the degree of methyl-esterification, and the main source of commercial pectins is apple pomace (low degree of methylation = 24%) and citrus peels (high degree of methylation = 74%).

Alginate as a natural polysaccharide is isolated from brown algae, and its monomers are α-L-guluronic acid and (1,4)-linked β-D-mannuronic acid [17]. This natural biopolymer is widely used for producing films, gels, suspensions, and emulsions [18]. About 30,000 metric tons of alginate is produced annually, originating from brown algae (genera Laminaria and Macrocystis) [19]. Alginate is renowned for its biocompatible and biodegradable properties as well as its low price. An important characteristic of alginate is the ability of functional groups (carboxyl and hydroxyl) of the G blocks to react with polyvalent cations (e.g., Ca^2+^, Al^3+^, and Fe^3+^) [20]. Among the divalent ions, calcium ions are commonly reacted with alginate to form a low solubility polymer. In general, G block longitude characterizes the ability and selectivity of the alginate in forming these interactions, whereas M and MG blocks are almost without selectivity. M and G blocks associate through ions to form a 3D structure (“egg-box”) [21,22]. This triggers an anion exchange process in which the water-soluble alginate exchanges its counter ions with Ca^2+^. This ionic crosslinking leads to cold-setting and heat-stable films. Due to the hydrophilicity of alginate films, they are crosslinked for improvement of their resistance in water, mechanical properties, and coherence [23].

Selecting convenient materials and packaging techniques are necessary for the maintenance of the product freshness and modality. Therefore, packaging materials which react with the foodstuffs and preserve them are of interest [2]. Active packaging is described as the packaging with components in the material or packaging empty space which improves the performance of the package [24]. Antimicrobial packaging has a great influence on the food packaging industry due to controlling the bacteria growth, lengthening the shelf-life, and ensuring the health of foodstuffs [25]. In this system, antimicrobial agents are embedded in the polymeric matrix. Biopolymers can be used for the construction of a biodegradable antimicrobial packaging. Essential oils [26]), plant extract [27], bacteriocin [28], enzymes [29], chitosan [30], organic acids [31], metallic nanoparticles [32], and chelating agents [33] are the antimicrobial agents which have been used in biodegradable polymers.

Various antimicrobial agents have been incorporated in the pectin films to create antimicrobial active packaging which prolongs the shelf-life and reduces the bacteria growth on the product surface [34]. For instance, Grau et al. [35] prepared pectin and apple puree based films with oregano, cinnamon, or lemongrass essential oil incorporated in them. Developed samples possessed antimicrobial properties for *E. coli* bacteria, and the oregano edible film had most efficiency against assayed bacteria. In the other study, Ravishankar et al. [36] incorporated cinnamaldehyde and carvacrol in apple puree and pectin films at 0.5%, 1.5%, and 3% (*w*/*w*) and evaluated their antibacterial activity. Results revealed that films including carvacrol have stronger antibacterial activity against *E. coli* and *S. enterica* in comparison to samples containing cinnamaldehyde.

On the ground of wide use of biopolymers and biocomposite in recent technologies, researchers are interested in developing mechanisms which are capable of healing and repairing the bio-based material after fracture, in order to restore the mechanical strength of primary material. The accustomed repairing methods of bio-based materials are quite complicated and can be expensive and time-consuming [37,38]. Hence, a novel healing method which employs a simple, safe, and environmentally friendly approach is highly desirable in order to bridge the damaged zones in bio-based materials by the formation of new covalent bonds.

In this study, healable and safely dissolvable alginate–pectin based biocomposites have been prepared. These films are applicable in the food packaging industry. Physico-chemical properties of all biocomposites were extensively investigated with mechanical and thermal analysis and wettability test. The morphological study was used to determine the structural properties. Tensile properties indicated that the fabricated samples have mechanical properties in the range of commercially available packaging films while possessing excellent healing efficiency. Biocomposite films exhibited highly hydrophobic properties, while thermal analysis showed that crosslinked biocomposite films possess higher thermal stability in temperatures below 120 °C. Biocomposite films exhibited antibacterial activity against various bacteria. Therefore, this study deals with the development of biodegradable films for foodstuff packaging application.

## 2. Materials and Methods

### 2.1. Materials

Pectin from the citrus peel (LM pectin, galacturonic acid, >74.0%) and lactic acid (ACS reagent, ≥85%) were obtained from Aldrich (Saint Louis, MO, USA). Medium range molecular mass sodium alginate (Manugel GHB, FMC Biopolymer, Ayrshire, UK, 37%(M)–63%(G)) was used as received. Ascorbic acid (99%) and calcium chloride dehydrate (99+%) were obtained from ACROS Organics (Geel, Belgium), while tri-sodium citrate dehydrate (294.10 g/mol, 99+%) was purchased from Merck (Darmstadt, Germany).

### 2.2. Preparation of Biocomposite Films

**Figure 1:**
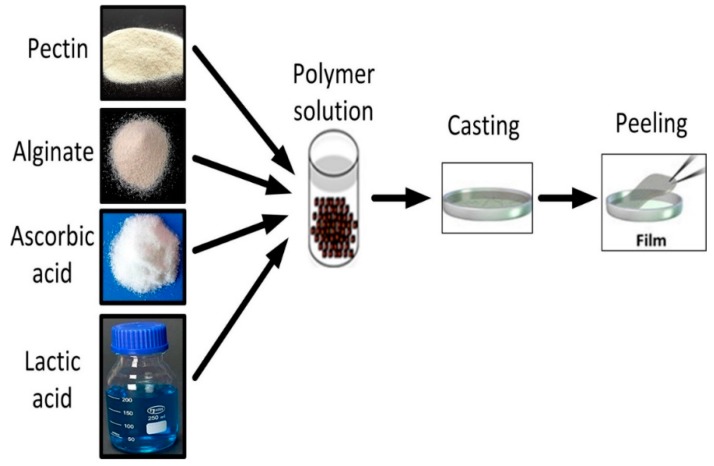
Schematic diagram explaining preparation of functionalized pectin–alginate films by solution casting method.

Aqueous alginate and pectin solutions containing 1 wt% of each biopolymer with mixing ratio of 1:1 was prepared by dissolving 0.7 g of pectin and 0.7 g of alginate together in 70 mL of deionized water and stirring for 4 h (500 rpm) using a magnetic stirrer at room temperature. Then, 5 wt% AA and 5 wt% LA, with reference to the total polymer weight, were added to the solution and stirred for another 4 h. The resulting solution was poured into a glass petri dish (diameter, 15 cm) and was dried in the oven for 24 h at 35 °C to form a thin film (Figure 1).

### 2.3. Crosslinking and Decrosslinking of Biocomposite Films

Samples were crosslinked in 2 wt% calcium chloride solution and then decrosslinked in 5 wt% sodium citrate solution. The crosslinking time of 2 min was observed to be sufficient for complete crosslinking of the biocomposite films. However, the decrosslinking time varied for different compositions and has been reported.

### 2.4. Healing of Biocomposite Films

**Figure 2:**
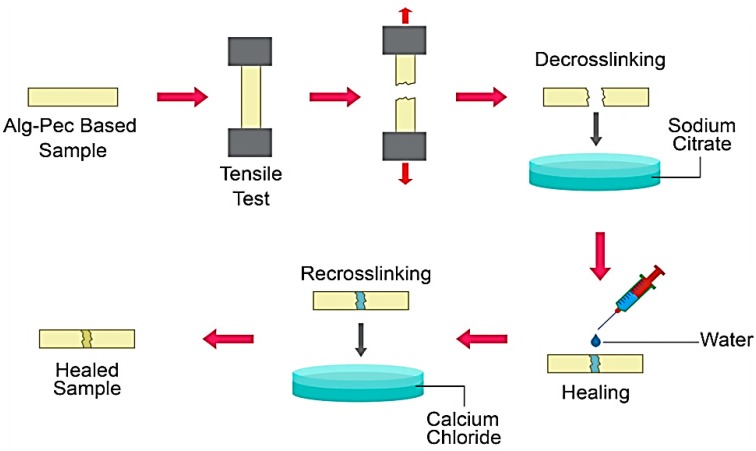
Healing process of alginate–pectin (Alg–Pec) based samples.

Separated strips of pectin-alginate based biocomposite films were healed and stuck together after the tensile test. Briefly, strips were decrosslinked in a solution of 5 wt% sodium citrate and then, after drying, a thin line of water applied on the edge of the films by a syringe. Then, the edge of the films were pressed together (Figure 2). Samples were crosslinked again in 2 wt% calcium chloride solution after drying.

### 2.5. FE-SEM Observation

Morphological information of biocomposite films were revealed with ultra-high resolution field emission scanning electron microscope (Model SU8010, Hitachi, Tokyo, Japan). Before imaging, the samples were coated with a thin layer of platinum (4–7 nm) using a Q150R S rotary-pumped sputter coating system (Quorum Technologies, Lewes, UK) at 2.5 KV before being observed to prevent electrostatic charging.

### 2.6. FT-IR Spectrometry

FT-IR spectra were recorded using a spectrometer (Model IS10, Thermo scientific, Waltham, MA, USA) to investigate the chemical composition and interactions of the biocomposite films. All spectra were recorded using a 0.4 cm^−1^ resolution with 32 scans per sample in the 4000 to 600 cm^−1^ wavelength region.

### 2.7. Hydrophilicity Analysis

The Water contact angle of the samples was measured with an optical device (Model 250, rame-hart instrument, Succasunna, NJ, USA). After fixing the samples on a support surface, the water contact angle was determined with the sessile drop method for 80 s. At least five measurements were performed on each sample and the volume of the water droplet was 2 ± 0.2 µL.

### 2.8. Thermogravimetric Analysis

The thermogravimetric analysis (TGA) is a thermoanalytical technique that provides a clear picture of the thermal stability and the interaction of polymer blend components. TGA was performed using a thermogravimeter (Model Q50, TA Instruments, New Castle, DE, USA). The weighed samples (6 mg) were heated with a temperature range of 25–700 °C at a rate of 10/min under the nitrogen flow 60 cm^3^ min^−1^ for the specimen and 40 cm^3^ min^−1^ for the balance.

### 2.9. Tensile Property

Mechanical characterization of alginate, pectin, and their biocomposite samples was carried out by tensile analyzer (Model 5982, Instron, Norwood, MA, USA) through stress-strain curves. Rectangular samples (10.00 × 2.00 mm^2^) were prepared and the tensile test was performed at rate of 2 mm/min. Tensile stress at the fracture point and elongation at rupture were determined. In addition, the tensile properties of healed samples were obtained. The tensile measurements were repeated 3 times and average values with standard deviation are provided. The healing efficiency (ƞ) is defined as the ratio of the tensile strength of the healed sample (σ healed) to that of pristine ones (σ pristine):

(1) $$\eta =\frac{\sigma \;\mathrm{healed}}{\sigma \;\mathrm{pristine}}$$

### 2.10. Antimicrobial Evaluation

Gram-negative *Escherichia coli* ATCC 25922, *Pseudomonas aeruginosa* ATCC 10145, and Gram-positive *Staphylococcus aureus* ATCC 29213 were employed to test biocomposite films antibacterial activity by disk diffusion method. All bacteria were cultured in nutrient broth (Merck, Darmstadt, Germany) overnight at 37 °C. After absorption of each culture on the nutrient agar (Merck, Darmstadt, Germany) for 15 min, prepared discs from biocomposite films (diameter = 6 mm) were placed on the agar plate. The Diameter of inhibition zone was measured after an overnight incubation at 37 °C for 18 h to determine the inhibitory activity of the biocomposite films.

### 2.11. Calcium Concentration

The measurements of the calcium concentration in the crosslinked samples were performed by inductive coupled plasma-optical emission spectrophotometer (Model Optima 8000, Perkin Elmer, Waltham, MA, USA). Composite films (about 10 mg) were dissolved in 20 mL of a 2 wt% sodium citrate solution and three measurements were carried out on each sample.

## 3. Results and Discussion

### 3.1. Dissolvability and Reusability Analysis

Dissolvability and reusability of casted films of pectin, alginate, and their biocomposites were analyzed by a series of crosslinking and decrosslinking processes (Table 1). Samples were crosslinked in 2 wt% calcium chloride solution and then decrosslinked in 5 wt% sodium citrate solution. It can be seen that alginate samples can go through the crosslinking–decrosslinking process multiple times without deterioration (>5 times), while they can fully dissolve in water after decrosslinking in less than 20 min. Similarly, alginate–pectin (Alg–Pec) and Alg–Pec–AA–LA samples have the ability to be crosslinked and then decrosslinked for several times while having the ability to completely dissolve in water in 10 min. In contrast, pectin samples did not possess the ability to be crosslinked after the first decrosslinking. Interestingly, these samples were able to be dissolved in the decrosslinking solution, while this was not the case for other samples and they had to be taken to a beaker containing water to start dissolving.

**Figure 3:**
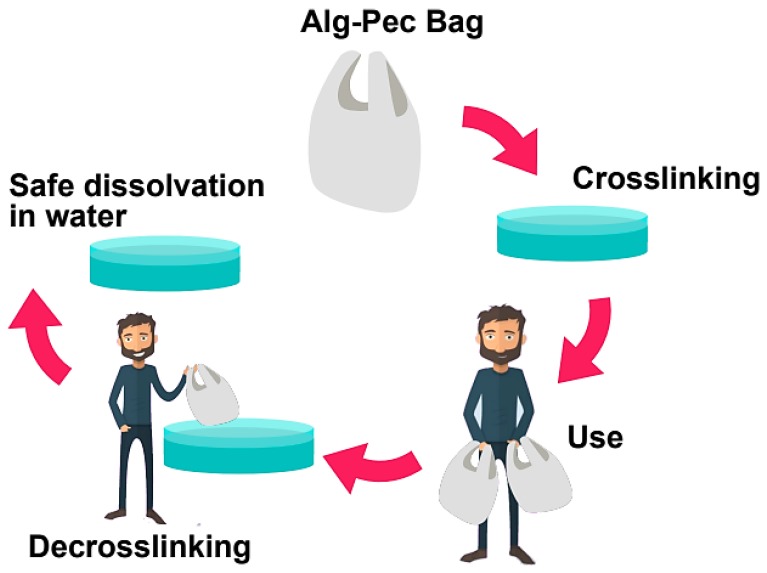
Potential utilization and disposal of Alg–Pec based films.

Potential utilization and safe disposal of the fabricated alginate–pectin based films are illustrated in Figure 3.

**Figure 4:**
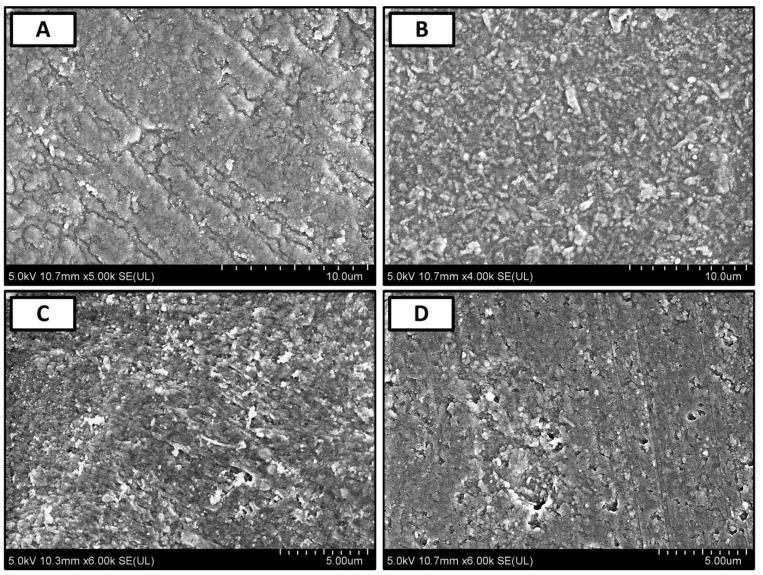
FE-SEM micrographs of (**A**) crosslinked alginate, (**B**) pectin, (**C**) Alg–Pec, and (**D**) Alg–Pec–AA–LA biocomposite films.

FE-SEM micrographs of crosslinked alginate, pectin, and their biocomposites are reported in Figure 4. As shown in Figure 4A, alginate films have a semi-rough surface with obvious patches of smooth surfaces, while high content of roughness can be observed all over the surface of the pectin films (Figure 4B). In the case of Alg–Pec biocomposite films (Figure 4C) and Alg–Pec–AA–LA (Figure 4D) it can be seen that the surface roughness in these samples are similar to the pectin samples. It has been reported that surface roughness has a direct effect on the hydrophilicity of material [39]. Hence, it is expected that an Alg–Pec biocomposite film possesses different degrees of hydrophilicity than pristine alginate film.

### 3.2. FT-IR Spectrometry

**Figure 5:**
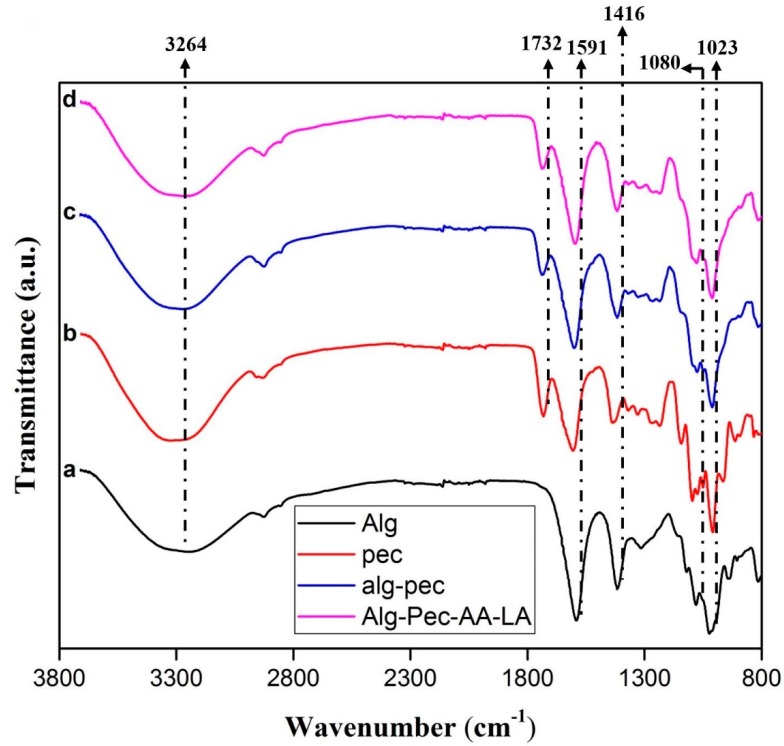
FT-IR spectra of crosslinked (**a**) alginate, (**b**) pectin, (**c**) Alg–Pec, and (**d**) Alg–Pec–AA–LA films.

The FT-IR spectra of crosslinked films of alginate, pectin, and their biocomposites are reported in Table 2 and Figure 5. For a pure pectin film, a wide peak between 3600 and 3000 cm^−1^ is attributed to the stretching of O–H because of hydrogen bonding interactions in the galacturonic acid [40]. Due to bending vibrations of CH, CH_2_, and CH_3_, a mildly sharp peak between 3000 and 2500 cm^−1^ is observed. Strong peaks at 1732 and 1605 cm^−1^ are assigned to the –CO of the methyl ester group (–COOCH_3_) and asymmetric stretching of the carbonyl group of the carboxylate ion (COO^−^), respectively, and a weaker peak can be seen at 1435 cm^−1^ due to the symmetric stretching of the carboxylate ion [40]. Stretching vibrations of C–O–C and C–C of the glucose ring appeared between 1360–800 cm^−1^ which is the “finger print” region and is specific for each compound [41,42].

In the Alg–Pec samples, the shifting of –OH and asymmetric stretching vibrations of COO^−^ peak from 3378 to 3300 cm^−1^ and from 1605 to 1601 cm^−1^, respectively, is due to the hydrogen bonding interactions between the polar groups of both polymers and polyglycerol. Actually, the shifting of the vibrational peaks to the lower wavenumbers is due to the mixing of the miscible Alg–Pec blend which leads to a balance between the enthalpy and entropy and consequently decreasing the free energy of the system. This result revealed that hydrogen bonding interaction between polymers in the blend system is more powerful in comparison to hydrogen bonding in the pure polymers [41,42].

### 3.3. Hydrophilicity Analysis

**Figure 6:**
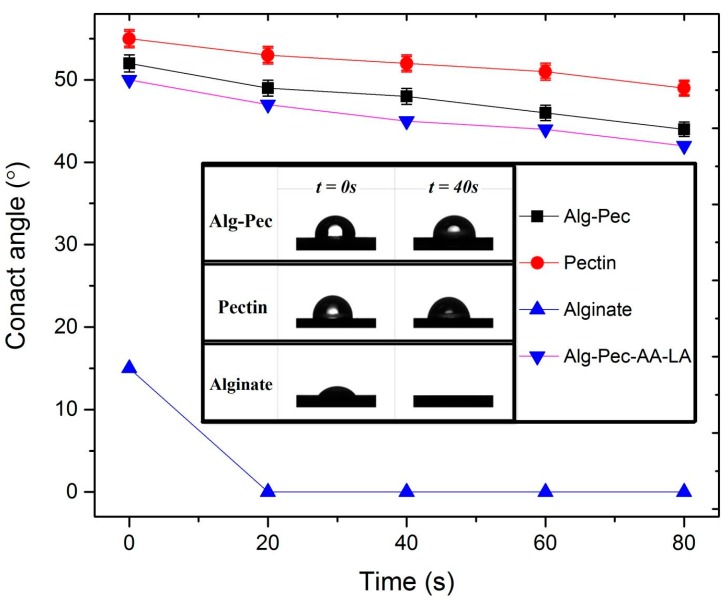
Water contact angle evaluation of alginate–pectin based films over time.

The water contact angle of biocomposite samples is shown in Figure 6. It was observed that the water contact angle values of Alg–Pec films decreased in the presence of alginate which is attributed to the hydrophilicity of Alg–Pec samples due to the hydrophilic functional groups such as hydroxyl, carboxylate, and ether in the alginate structure. In addition, as described in the morphology analysis section, pectin and Alg–Pec films possess higher surface roughness in comparison to alginate films; hence, they have higher values of contact angle. It has been reported that surface roughness has a direct effect on the contact angle of surfaces, and an increase in surface roughness will lead to an increase in hydrophobicity of surfaces [39]. Moreover, the water contact angle decreased during measurements due to the droplet absorption and extension. Similar results were reported for pectin based biocomposites [43,44] and other biopolymers [45]. Alginate–pectin samples functionalized by AA and LA had an insignificant increase in their surface hydrophilicity (50°) in comparison to pristine Alg–Pec samples (52°), indicating that an incorporation of AA and LA did not have an effect on surface properties of these samples.

### 3.4. Tensile Property

Mechanical properties of the alginate, pectin, and their biocomposite films are presented in Table 3. It can be seen that the alginate film exhibit higher tensile strength in comparison to the pectin and Alg–Pec films, attributed to the stronger molecular structure and lower thickness. Oakefull et al. [46] investigated the interaction between alginate and pectin through polyguluronate blocks and methyl ester region that caused a rigid packed structure.

Tensile strength and elongation at break of alginate and pectin samples were decreased after crosslinking perhaps due to the fact that a crosslinking reaction occurred in an aqueous solution containing a crosslinking agent. Therefore, some water stayed in the interchain space in the network. Indeed, crosslinked dry samples are thicker in comparison to uncrosslinked ones. Russo et al. [47] reported a study about the crosslinking of alginate–polyglycerol with calcium ion that confirms the mentioned hypothesis. The crosslinked samples lost their flexibility due to the reduction of chain mobility resulting from crosslinking reaction. It should be noted that differences are significant considering the experimental errors. Conversely, a small increase was observed in the tensile value of crosslinked Alg–Pec based films compared to the same uncrosslinked film. This could be attributed to the fact that chain entanglement and connection parts in the blended film reduced the swelling. Incorporation of AA and LA led to a small decrease in value of tensile strength and elongation of Alg–Pec films.

### 3.5. Healing Property

**Figure 7:**
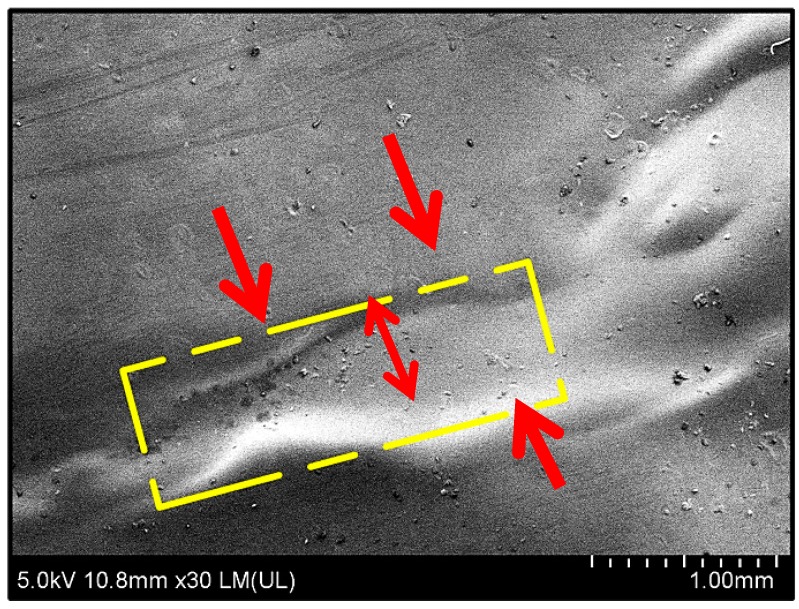
FE-SEM micrographs of healed Alg–Pec films. Arrows are pointing at healed regions and boundaries.

In order to evaluate the heal-ability of these films, rectangular shaped strips of biocomposite films of Alg–Pec with a size of 10.00 × 2.00 mm^2^ were pulled by the tensile machine until failure and then un-crosslinked by being dipped in a 2% sodium citrate solution for 5 min. After drying, both pieces of the film were patched together by applying a thing line of water, which acted like glue. A FE-SEM micrograph of these healed films is illustrated in Figure 7. A smooth surface can be seen at the patched line and intersection of both films, which indicates excellent adhesion between the surfaces of both films.

Healing efficiency of alginate and alginate–pectin based films is tabulated in Table 4. It can be seen that the healing process is considered to be highly effective in providing the strong bonding between two ruptured strips. This is due to the fact that the line of water applied to the decrosslinked area of the sample dissolves the surface in contact on both samples and this surface provides new bonding between samples after drying, which leads to impressive healing efficiency of the sample. A pristine alginate film with 92.5% healing efficiency pretty much sustains its mechanical strength, even after a process of crosslink–decrosslink. However, the pristine pectin film lost its rigidity after the decrosslinking process. Hence, the healing efficiency of this sample was not obtained. Healing efficiency of alginate–pectin based samples with (87.6%) or without AA and LA (84.1%) revealed high healing capacity of these samples, indicating the high potential of these samples to be utilized in packaging applications.

**Figure 8:**
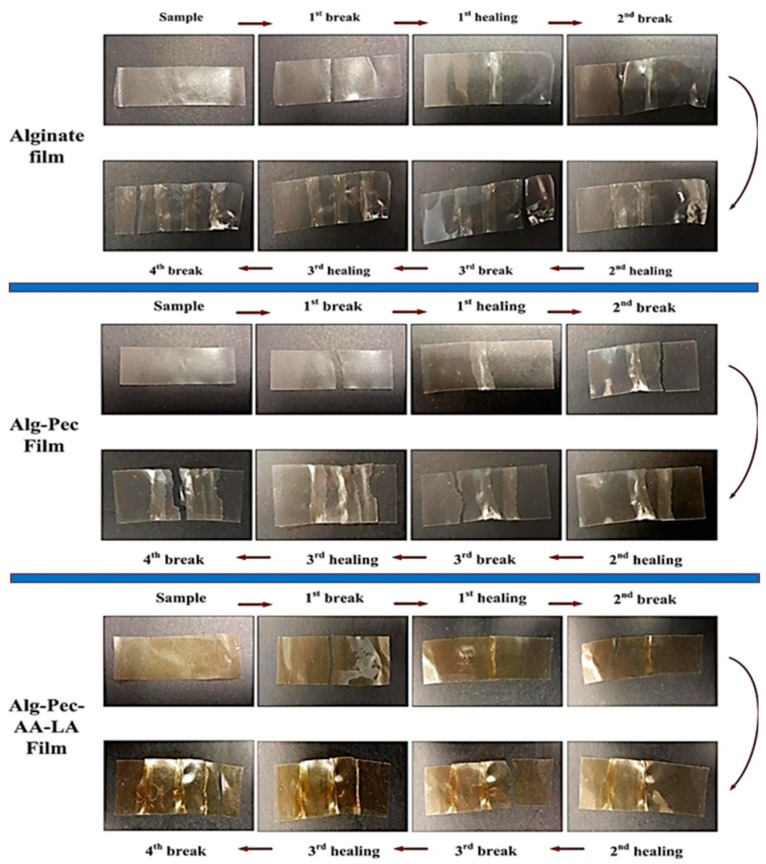
Repeated healing of alginate and alginate–pectin based biocomposites.

In order to examine the repeatability of the healing process on alginate–pectin based films, tensile strength was obtained from samples after every healing. As shown in Table 4, the value of tensile strength gradually reduces after every healing. For instance, in pristine alginate film, the value of the tensile strength decreased from 26 MPa to 16.4 after three healings. The noteworthy and interesting observation was that these samples never break from the healed line, indicating that the healing process completely bonds the separated pieces of the film after every breakage (Figure 8). Hence, the reduction in tensile strength of the films was associated with elongation of the polymer chains which reduces the overall strength of the samples. Even though the mechanical properties of these films reduced after every healing, the heal-ability of these films could be beneficial for various applications, since it is favorable for the environment and eliminates the costs associated with the replacement.

**Figure 9:**
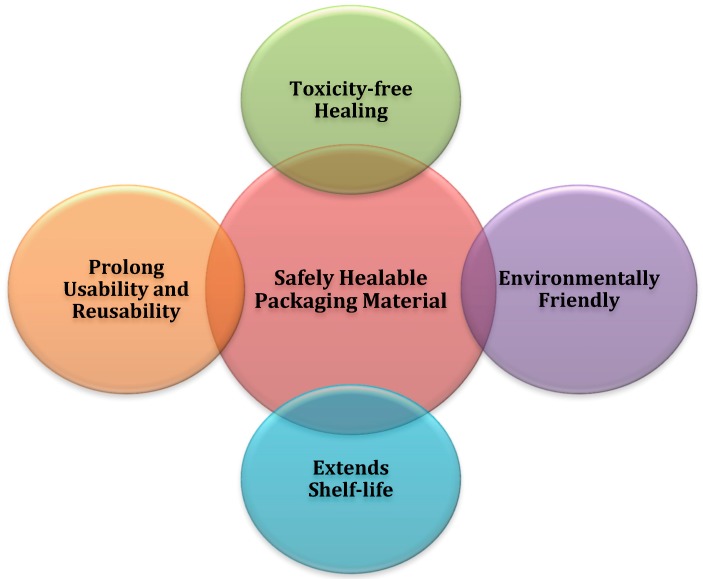
Features and functions of safely healable packaging materials.

Polymeric packaging can be damaged by the mechanical, chemical, and thermal stimulus [37]. Each year an overwhelming amount of foodstuff with damaged packaging is described due to deterioration caused while transportation. Healable polymeric packaging material will effectively prolong the usability and reusability of the packages, which directly reduces the food wastage and plastic pollution (Figure 9). Since progress in healable materials is important for developed societies, several researchers proposed healing mechanisms for packaging applications. For instance, Andersson et al. [48] investigated specific microcapsules as self-healing agents in paperboard coatings. The microcapsules were coated on the surface of paperboard and the healing agent was released while external forces were applied during creasing and folding operations. Results indicated that plasticization of the coating hindered the crack propagation while releasing the hydrophobic healing agent from the microcapsules increased the hydrophobicity and consequently coating properties were preserved. 

### 3.6. Thermogravimetric Analysis

**Figure 10:**
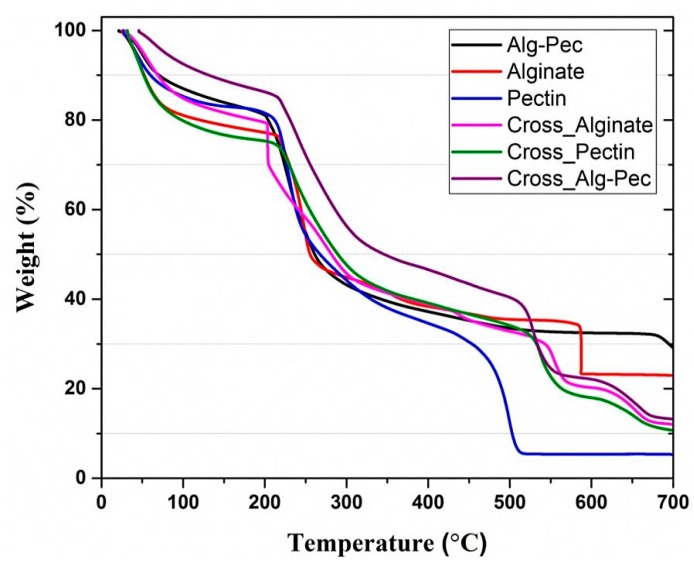
Thermogravimetric analysis (TGA) curves of alginate, pectin, and their biocomposites before and after crosslinking.

Figure 10 represents TGA curves of alginate, pectin, and their biocomposites before and after crosslinking. The weight of the samples decreased in the range of 25–120 °C due to the removal of moisture, followed by sharp weight loss at ca. 220 °C because of the biopolymer degradation. Lower rate of weight loss can be seen in crosslinked samples from 25 to 120 °C, indicating that crosslinking leads to an increase in thermal stability at temperatures below 120 °C and delays the degradation temperature. The removal of water has caused the weight loss of crosslinked samples. Since the weight loss below 120 °C is associated with the elimination of retained water, the crosslinked outer layer of the films provides a barrier that reduces the amount of evaporation of these water molecules and therefore diminishes the rate of mass loss.

Thermal properties of biocomposite samples are reported in Table 5. Crosslinked films of Alg–Pec show the lowest value of weight loss at 100 °C which is important for packaging applications. This indicates the stability of these samples and they can be used in the packaging of hot food and beverages. In addition, the value of weight loss at 100 °C reduced for both alginate and Alg–Pec samples after crosslinking, while the value of temperature at 5% weight loss increased. This indicates the notable effect crosslinking on improving the thermal stability of these samples in temperatures below 100 °C. In contrast, crosslinking did not lead to an improvement of thermal properties of pectin samples.

Interestingly, studying the percentage of residual matter at 700 °C revealed that crosslinked samples of alginate and Alg–Pec had lower values of residue char in comparison to their pristine samples, while the opposite was true for pectin samples. This indicates that the existence of Ca ions between the bonds of alginate leads to further degradation and reduces the thermal stability at high temperatures in comparison to the pristine alginate sample. However, it was observed that temperature values at maximum weight loss rate of crosslinked samples of pectin and Alg–Pec were lower in comparison to their pristine samples, indicating that crosslinking improved the thermal stability of samples containing pectin at high temperatures. It must be mentioned that the TGA result of Alg–Pec–AA–LA samples was not included in the discussion since no major variation in the result was observed in this sample in comparison to the pristine Alg–Pec sample.

### 3.7. Antimicrobial Evaluation

Different bacteria were employed to test the antimicrobial activities of the alginate–pectin biocomposite films containing AA and LA by disk diffusion method. As shown in Table 6, biocomposite films were more effective against *E. coli* and *S. aureus*, while no antibacterial activity was observed in the pristine Alg–Pec sample or Alg–Pec samples containing either AA or LA. It has been reported that AA has effective antibacterial activity when it is used in combination with LA. In a study conducted by Tajkarimi et al. [49], it was reported that the incorporation of 0.4% AA and 0.2% LA inhibited the growth of *E. coli* O157:H7 in carrot juice, indicating their potential to be utilized as preservative. AA absorbs the oxygen and prevents reaching of oxygen to *E. coli* O157:H7, while the synergistic effect of AA and LA can be same as the synergetic effect of air, oxidizing agents, transition metal ions against enteroviruses [49].

Varying antimicrobial effects of the biocomposite films were attributed to the different types of test bacterial pathogens used for antimicrobial evaluation, particularly in terms of the pathogen microbial structures. *P. aeruginosa* is known to be a heavy producer of extracellular matrices. Such matrixes blocks and resists the movement of the antimicrobial component of the film towards the cells themselves [50]. Conversely, as *S. aureus* is the only gram-positive bacteria tested, it has a peptidoglycan cell wall that enables it to avoid lysis upon damage of its sensitive membrane [51]. Being gram-negative, *E. coli* and *P. aeruginosa* both posess outer membranes that coat their cells and enable them to further repel antibacterial components away from the cells. These findings point towards the antibacterial potential of the biocomposites rather than of its mode of action.

Non-polar components of the film, however, may also be antibacterial and hence only be visually active upon direct cell contact with the film itself. This is because the disk diffusion assay employed here depends largely on the polarity, size, and chemical structure of the tested compounds as the agar medium comprises largely of water. *S. aureus* and *E. coli* are both inhibited (zones) by the film away from the edges of the disk due to diffusion of antibacterial compounds of which at varying concentrations, have a stronger effect against *E. coli* than *S. aureus* at a lower concentration (further away from the disk). However, *P. aeruginosa* is resistant against the film.

### 3.8. Calcium Concentration

The calcium content of alginate, pectin, and their biocomposite films is shown in Table 7. Alginate samples have more calcium content followed by composite and pectin samples. It should be noted that differences are far above the statistical error (1%). These results are consistent with previous results about the swelling and mechanical properties of the samples and augment the hypothesis that alginate film was crosslinked more effectively in comparison to pectin sample. Same results were observed in studies conducted by Sriamornsak et al. [52] and Da Silva et al. [53] in which alginate films had more calcium in comparison to pectin films.

## 4. Conclusions

In this study, we have prepared active, healable, and safely dissolvable alginate–pectin based biocomposites which have potential to be used in the food packaging industry. The morphological study revealed the rough surface of these biocomposite films, while antibacterial analysis indicated effective activity of functionalized films against various bacteria. Mechanical properties indicated that the fabricated biocomposite films have mechanical properties in the range of commercially available packaging films while possessing excellent healing efficiency. On the grounds of extensive use of biopolymers and biocomposites in various applications, the ability to recover the mechanical and physical properties of pristine materials after damage is an attractive feature. The fabricated multifunctional biocomposites with simple, low-cost, and environmentally friendly heal-ability properties proposed in this study have tremendous potential to be utilized in various applications such as packaging material.

## Figures and Tables

**Table 1:** Dissolvability and reusability analysis of pectin, alginate, and their biocomposite films.

Sample	Thickness (mm)	Crosslinking Time (min)	Decrosslinking Time (min)	Crosslinking Repeatability (times)	Decrosslinking Repeatability (times)	Complete Dissolving after Decrosslinking (min)
Alginate	0.043 ± 0.008	2	7	>5	>5	15
Pectin	0.038 ± 0.15	2	1	-	-	2
Alg–Pec	0.043 ± 0.013	2	6	>5	>5	10
Alg–Pec–AA–LA	0.056 ± 0.01	2	6	>5	>5	9

**Table 2:** FTIR-ATR main vibrational frequencies of pectin–alginate based films.

Band Frequency (cm^−1^)				
Sample	–OH	COO^−^ (asym)	COO^−^ (sym)	COOCH_3_
Pectin	3378	1605	1435	1732
Alginate	3264	1591	1416	-
Alg–Pec	3300	1601	1417	1738
Alg–Pec–AA–LA	3282	1594	1417	1736

**Table 3:** Tensile properties of the alginate, pectin, and their biocomposite films before and after crosslinking ^a^.

Sample	Crosslinking Time (min)	Thickness (mm)	Tensile (MPa)	Elongation (%)
Alginate	0	0.024 ± 0.01	29.1 ± 3.1	8.16 ± 0.9
Alginate	2	0.043 ± 0.008	26.05 ± 4.7	7.3 ± 1.8
Pectin	0	0.035 ± 0.012	18.7 ± 3.2	3.9 ± 0.5
Pectin	2	0.038 ± 0.15	16.8 ± 2.4	3.5 ± 1.2
Alg–Pec	0	0.033 ± 0.014	20.1 ± 1.7	11.8 ± 1.5
Alg–Pec	2	0.043 ± 0.013	23.4 ± 0.9	9.7 ± 2.6
Alg–Pec–AA–LA	0	0.039 ± 0.14	19.3 ± 2.7	10.9 ± 1.3
Alg–Pec–AA–LA	2	0.048 ± 0.01	22.7 ± 1.8	9.1 ± 0.8

^a^ Errors represent standard deviation for three independent measurements.

**Table 4:** Healing efficiency of crosslinked alginate and alginate–pectin based films.

Sample	Crosslinking Time (min)	Thickness (mm)	Tensile (1st healing) (MPa)	Elongation (1st healing) (%)	Healing Efficiency (1st healing) (%)	Tensile (2nd healing) (MPa)	Elongation (2nd healing) (%)	Tensile (3rd healing) (MPa)	Elongation (3rd healing) (%)
Alginate	2	0.043 ± 0.008	24.1 ± 2.4	6.5 ± 1.1	92.5	20.5 ± 1.8	5.1 ± 0.3	16.4 ± 1.3	3.8 ± 0.4
Alg–Pec	2	0.043 ± 0.013	20.5 ± 1.7	9.1 ± 1.5	87.6	15.8 ± 1.5	7.4 ± 1.2	12.1 ± 0.9	4.9 ± 0.5
Alg–Pec–AA–LA	2	0.048 ± 0.01	19.1 ± 1.8	8.5 ± 0.8	84.1	14.5 ± 0.7	6.1 ± 0.5	10.7 ± 1.1	4.2 ± 0.7

**Table 5:** Thermal stability parameters of alginate, pectin, and their biocomposite films before and after crosslinking.

Sample	Temperature at 5% Weight Loss (°C)	Weight loss at 100 °C (%)	Residual Matter at 700 °C (%)	Temperature at Maximum Weight Loss Rate (°C)
Alginate	41.05	18.96	22.95	249, 587
Pectin	42.51	14.72	5.33	228, 498
Alg–Pec	49.10	12.98	29.10	229, 690
Crosslinked Alginate	51.18	15.13	12.07	203, 553
Crosslinked Pectin	40.97	20.17	10.67	232, 537
Crosslinked Alg–Pec	78.11	7.48	13.21	240, 527

**Table 6:** Antimicrobial activity of functional biocomposite films.

Bacteria	Diameter of Zone of Inhibition (mm)
*E. coli*	9.0 ± 1.5
*P. aeruginosa*	6.0 ± 2.0
*S. aureus*	8.0 ± 1.0

**Table 7:** Calcium content of pectin, alginate, and their biocomposite films.

Sample	Calcium Content (mg/g sample)
Alg	32.18
Pec	22.7
Alg–Pec	27.8
Alg–Pec–AA–LA	30.78
